# Therapeutic strategies for tongue musculature: a systematic literature review

**DOI:** 10.1590/2317-1782/e20240089en

**Published:** 2025-01-20

**Authors:** Andressa Colares da Costa Otavio, Arthur Cherem Netto Fernandes, Maria Eduarda Pollachinni Andrade, Brenda Barros Dias, Erissandra Gomes, Marco Aurélio Vaz

**Affiliations:** 1 Faculdade de Odontologia, Universidade Federal do Rio Grande do Sul – UFRGS - Porto Alegre (RS), Brasil.; 2 Programa de Pós-graduação em Saúde da Criança e do Adolescente, Universidade Federal do Rio Grande do Sul – UFRGS - Porto Alegre (RS), Brasil.; 3 Universidade Federal do Rio Grande do Sul – UFRGS - Porto Alegre (RS), Brasil.; 4 Departamento de Cirurgia e Ortopedia, Faculdade de Odontologia, Universidade Federal do Rio Grande do Sul – UFRGS - Porto Alegre (RS), Brasil.; 5 Laboratório de Pesquisa do Exercício, Escola de Educação Física, Fisioterapia e Dança, Universidade Federal do Rio Grande do Sul – UFRGS - Porto Alegre, RS, Brasil.

**Keywords:** Tongue, Exercise, Myofunctional Therapy, Physiology, Speech, Language and Hearing Sciences

## Abstract

**Purpose:**

To analyze the different therapeutic strategies prescribed in orofunctional rehabilitation of the tongue musculature.

**Research strategies:**

Regional Portal of the Virtual Health Library for Latin America and the Caribbean, Embase, PubMed/MEDLINE, Scientific Electronic Library Online, SciVerse Scopus and Cochrane databases were consulted, with the descriptors "exercise therapy” OR “physiology” OR “musculoskeletal physiological phenomena” OR “digestive system and oral physiological phenomena” AND “speech therapy” OR “myofunctional therapy” OR “speech language pathology” AND “tongue”. Studies indexed until October 5, 2023, were included.

**Selection criteria:**

Studies with an interventionist design with exercises for tongue musculature were included.

**Data analysis:**

Three reviewers selected, extracted and tabulated the information from the studies. The PEDro scale was used to measure the studies’ methodological quality.

**Results:**

1.036 studies were found, and 18 were included in this review. The samples varied between 16 and 148 subjects, aged between 4 and 95 years. Only seven studies clearly described the exercises execution, and the number of sets, repetitions, and contraction duration. Fourteen studies clearly defined the exercises’ objectives. The average score of the PEDro Scale analysis was 6.9, and 56.25% of the studies scored ≥7.

**Conclusion:**

There is a lack of a clear description of the exercises’ goals and their clinical indications, which can lead to confusion and inadequate prescription. Future studies will need to provide a clear description of the outcomes, in order that we can define, according to the exercises and training program specificity, what the effects of different training methodological parameters in this musculature are.

## INTRODUCTION

The tongue is a muscular organ covered by mucosa, located in the oral cavity and composed of extrinsic and intrinsic musculature. The extrinsic musculature consists of the palatoglossus, the hyoglossus, styloglossus and genioglossus muscles. The latter originates in a bone structure, and is responsible for moving the tongue. The intrinsic musculature, consisting of the upper longitudinal, lower longitudinal, transversal and vertical muscles, has its origin and insertion in the tongue and changes its shape during tongue function^(1,2)^. Although anatomically the tongue muscles are studied separately, functionally there are some overlaps^(2)^.

There are several peculiarities among the tongue muscles. There are regional differences in the fiber type distribution of the extrinsic muscles, groupings of the different types, variability in their size, fibers’ division and interconnection, and abundance of loose connective tissue^(2)^. The tongue’s motor units, described in a ratio of 25 muscle fibers per motor neuron^(3)^, are small and concentrated in the anterior third, with 71% of type II fast-twitch fibers, while in the posterior third, 66% are type I fibers^(4)^. The intrinsic muscles, on average, contain less type I fibers (42%) in the blade than in the body (58%) and at the base (58%)^(2)^. These peculiarities make the function of the tongue muscles very complex, thereby increasing the difficulty for their rehabilitation.

The biomechanics of the tongue has some particularities, as the tongue does not exert significant external force, but continuously remodels itself to adequately performing its functions. The tongue blade has a smaller number of slow-twitch fibers, which seems convenient for performing fine motor tasks. Similarly, the abundance of loose connective tissue seems relevant for changes in shape. In the absence of bones, the muscles themselves provide the supports or pillars on which they mechanically interact, and this is probably because a rigid support conflicts with fine movement^(2)^.

The tongue performs unique movements during speech, chewing and swallowing; however, the anatomical specializations underlying these movements are still largely unknown^(1)^. The effectiveness of a therapy for tongue musculature requires an adequate exercise prescription. However, studies with a detailed description of the techniques are scarce^(5)^. Apparently, there is no clarity or unanimity about the parameters used for the exercise prescription in this musculature rehabilitation.

## PURPOSE

Therefore, the objective of this systematic literature review was to define the parameters (number of sets and repetitions, frequency, isometric time, rest time and therapy time) of the exercises prescribed in speech therapy intervention for tongue muscles. Secondarily, we sought to analyze the quality of interventionist studies in the area.

## RESEARCH STRATEGY

This systematic literature review was conducted according to the instructions of Cochrane Collaboration^(6)^ and PRISMA Guideline (Preferred Reporting Items for Systematic Reviews and Meta-Analysis)^(7)^. The protocol number registered in PROSPERO is CRD42020186283.

For the formulation of the proposal, the PICOS strategy was used: Participant - individuals of any age; Intervention - tongue muscles; Comparison - absence of comparison group; Outcome - parameters prescribed in the exercises for the tongue musculature; Studies - randomized or not – clinical trials, giving rise to the following structured question: What is the level of evidence for the exercises and parameters of tongue muscle training prescribed by speech therapists in the oromyofunctional rehabilitation of the tongue muscles?

The search strategy was initially established for the PubMed database, using the keywords identified in the Health Sciences Descriptors (HSD) related to the exposure of interest and results: "exercise therapy” OR “physiology” OR “musculoskeletal physiological phenomena” OR “digestive system and oral physiological phenomena” AND “speech therapy” OR “myofunctional therapy” OR “speech language pathology” AND “tongue". The Boolean operator OR was used to combine the terms in each PICO concept; the AND operator was used to combine the different concepts of the PICOS (participant, exhibition, outcome). A sensitive search strategy was adapted for the other databases: Embase, Latin American Literature of Health Sciences of the Americas and Caribbean - LILACS, Scopus, Cochrane and The Scientific Electronic Library Online - SciELO. The complete search strategy, with the terms used for the databases, are described in Table 1. Studies indexed until October 5, 2023, were included. The database results were cross-checked to locate and eliminate duplicates.

**Table 1:** Databases and word combinations

Databases	Search descriptors	Number of articles Date
Cochrane Central Register of Controlled Trials	(exercise therapy or physiology) OR (musculoskeletal physiological phenomena) OR (digestive system and oral physiological phenomena) AND (speech therapy) OR (myofunctional therapy) OR (speech language pathology) AND (tongue)	79 October 5, 2023
Cochrane Reviews	(exercise therapy or physiology) OR (musculoskeletal physiological phenomena) OR (digestive system and oral physiological phenomena) AND (speech therapy) OR (myofunctional therapy) OR (speech language pathology) AND (tongue)	4 October 5, 2023
Embase	(exercise therapy or physiology) OR (musculoskeletal physiological phenomena) OR (digestive system and oral physiological phenomena) AND (speech therapy) OR (myofunctional therapy) OR (speech language pathology) AND (tongue)	171 October 5, 2023
BVS Regional Portal	(exercise therapy or physiology) OR (musculoskeletal physiological phenomena) OR (digestive system and oral physiological phenomena) AND (speech therapy) OR (myofunctional therapy) OR (speech language pathology) AND (tongue)	0 October 5, 2023
Pubmed	(exercise therapy or physiology) OR (musculoskeletal physiological phenomena) OR (digestive system and oral physiological phenomena) AND (speech therapy) OR (myofunctional therapy) OR (speech language pathology) AND (tongue)	654 October 5, 2023
Scielo	(exercise therapy or physiology) OR (musculoskeletal physiological phenomena) OR (digestive system and oral physiological phenomena) AND (speech therapy) OR (myofunctional therapy) OR (speech language pathology) AND (tongue)	0 October 5, 2023
Scopus	(exercise therapy or physiology) OR (musculoskeletal physiological phenomena ) OR (digestive system and oral physiological phenomena ) AND (speech therapy) OR (myofunctional therapy) OR (speech language pathology) AND (tongue)	128 October 5, 2023

## SELECTION CRITERIA

Studies with an interventional design (randomized clinical trial and non-randomized clinical trial - quasi-experimental), which presented some exercise for the tongue musculature, were included. No language or date restrictions were applied. Observational articles, comments, letters, book chapters, editorials, communications, opinions, literature reviews, systematic reviews, conference abstracts, duplicate studies, intervention studies in reports or case series were excluded. Studies on syndromes, metabolic diseases and other basic features were also excluded. Studies not available were excluded. The review studies were read to see if any studies, not found in the search phase, could be included.

In this review, studies that prescribed exercise for the tongue musculature were considered. The main outcome of this review was the parameters used in the exercise prescription for this musculature.

## DATA ANALYSIS

Three reviewers independently (ACCO, ACNF, MEPA) analyzed the titles and abstracts, selecting those that would meet the eligibility criteria. Disagreements were discussed among the reviewers. Two reviewers independently (ACCO, ACNF) read articles considered eligible or uncertain in full, and selection criteria for inclusion were applied. The reasons for excluding the evaluated full texts were recorded. Next, three reviewers (ACCO, ACNF, MEPA) extracted and tabulated information regarding authors, year of publication, objective, methodological design, participants’ number and characteristics, exposure characteristics, outcome measures and main results.

Two authors, using the PEDro Physiotherapy Evidence Database Scale^(8)^, performed a blind and independent evaluation of the studies’ quality. This scale consists of eleven assessment items: specified eligibility criteria; random allocation to groups; secret allocation; similar groups; blinded participants; blinded therapists; blinded evaluators; result obtained in more than 85% of the initial sample; treatment intention; statistical comparisons; precision and variability measures^(8)^.

## RESULTS

### Results summary

The included studies were moved to data extraction, following a standard form in Google Drive®. These data were first summarized in spreadsheets, according to the nature of the outcome measures. For the results’ quantitative measures, the mean values, frequency, and standard deviations were recorded, whenever possible. Finally, the data were grouped into tables. We tried to summarize the data so that there was uniformity in the presentation. No meta-analysis was performed due to the data heterogeneity. It should be noted that this review was not intended to assess the effects of the exercises, but rather to characterize them. Thus, only the data directly related to the purpose of this review were tabulated in a synthesized way.

### Analysis of selected studies

**Figure 1:**
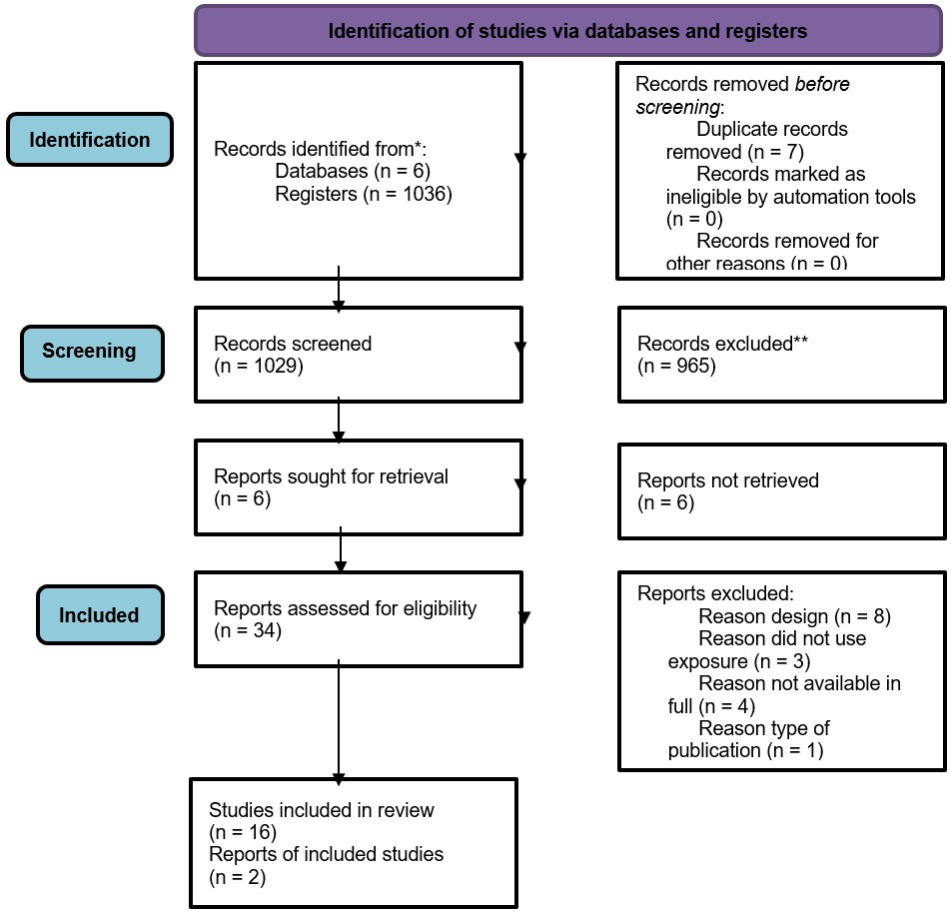
Flow diagram of article selection process

A total of 1,036 articles were found from the consulted databases. After removing duplicate records, 1,029 records remained. In analyzing the titles and abstracts, 965 studies were eliminated. Therefore, 34 studies met the inclusion criteria for full text review. After reading the studies in full, 16 articles were excluded according to the reasons described in Figure 1, and eighteen^(9-26)^ were included as they met the inclusion criteria (Figure 1).

*Consider, if feasible to do so, reporting the number of records identified from each database or register searched (rather than the total number across all databases/registers)

**If automation tools were used, indicate how many records were excluded by a human and how many were excluded by automation tools

From: Page et al.^(27)^. For more information, visit^(28)^: http://www.prisma-statement.org/

### Characteristics of included studies

The characteristics of the studies and participants are described in Table 2. The eighteen studies were carried out in eight countries, published between 2005 and 2023. In the last ten years, thirteen of these studies were published, seven in the last five years. The studies’ sample size varied between 16 and 148 subjects, aged between four and 95 years, with a large heterogeneity in their populations. Five studies evaluated patients with head and neck cancer and/or chemotherapy^(11,14,16,23,26)^; five evaluated patients with Apnea Syndrome and Obstructive Sleep Hypopnea^(10,17,20,24,25)^; two in anterior open bite^(9,18)^; two in healthy adults^(12,13)^; one in healthy elderly^(21)^; one in orthognathic surgery patients^(19)^ and two in patients with dysarthria after stroke^(15,22)^ (Table 2).

**Table 2:** Characteristics of included studies

Author/Year/Country	Background	Study Objective	n	Participants
Degan and Puppin-Rontani, 2005^(9)^	Children with anterior open bite with pacifier sucking habit	To study the effects of the association of OMT and the removal of sucking habits in the rehabilitation of swallowing and tongue rest	20	Pacifier and baby bottle removal group: n: 10
Brazil				
				OMT group: n: 10
				Age: 4 years to 4 years and 8 months both groups
				Sex: -
Guimarães et al., 2009^(10)^	Adults with moderate OSAHS	To determine the impact of oropharyngeal exercises in patients with moderate OSAHS	31	Placebo group: n: 15 | Age: 25-65 years (mean 47.7 SD: 9.8)
Brazil				
				Sex: 11 (73%) male, 4 (27%) female
				Exercise group: n: 16 | Age: 25-65 years (mean 51.5 SD: 6.8)
				Sex: 10 (63%) male, 6 (37%) female
Carnaby-Mann et al., 2012^(11)^	Adults with head and neck cancer	To evaluate the benefits of a set of exercises on the composition and function of swallowing muscles for patients with head and neck cancer who are undergoing radiation and chemotherapy	58	Usual care group: n: 20 | Age: - (average 54 SD: 11.3)
United States				
				Sex: 15 (75%) male, 5 (25%) female
				Placebo group: n: 18 | Age: - (mean 60 SD: 12.2)
				Sex: 11 (61.11%) male, 7 (38.89%) female
				High intensity group: n: 20 | Age: - (mean 59 SD: 10.4)
				Sex: 18 (90%) male 2 (10%) female
Kothari et al., 2011^(12)^	Healthy adult	To test the possible influence on tongue-training performance and motor learning of the natural ability to roll the tongue and modulations of tongue-training parameters by alteration of tongue-task timing.	44	Study 1: n: 29 | Aged 21–29 years
Denmark				
				Group with the natural ability to roll their tongue: n: 15
				Sex: 4 men, 11 women
				Group without the natural ability to roll their tongue: n: 14
				Sex: 3 men, 11 women
				Study 2: n: 15 | Age: 20–60 years
				Sex: 7 men, 8 women
Clark., 2012^(13)^	Healthy adults without history of speech or swallowing deficits	The objectives of Phase I (specific tongue training) include identifying the presence of a therapeutic effect and estimating its size	25	Age: 20-57 years (average 29.8 years)
United States				
				Sex: 3 male, 22 female
Lazarus et al., 2014^(14)^	Adults with head and neck cancer	To study the effects of tongue strengthening training associated with traditional exercises versus only traditional exercises	23	Study group: n: 12 | Age: - (mean 62.3 SD: 8.06)
Turkey				
				Control group: n: 11 | Age: - (average 61.7 SD: 7.27)
				Sex: 22 (96%) male, 1 (4%) female
Mackenzie et al., 2014^(15)^	People with chronic post-stroke dysarthria	To examine operational feasibility of the programme; participants’ views and speech intelligibility, communication effectiveness and tongue and lip movement at four points	39	Group A (intervention without NSOMExs): n: 20 | Age: - (mean 67.95 SD: 12.10)
UK				
				Sex: 14 (70%) male, 6 (30%) female
				Group B (intervention with NSOMExs): n: 19 | | Age: - (mean 62.80 SD: 12.52)
				Sex: 12 (63%) male, 7 (37%) female
Nuffelen et al., 2015^(16)^	Adults: head and neck cancer, dysphagia and chemotherapy	To investigate the effects of resistance levels on tongue muscle strength and swallowing function	51	Study in progress
Belgium				
Diaféria et al., 2017^(17)^	Adults, men with OSAHS	To assess the effect of myofunctional therapy on adherence to CPAP	100	Placebo: n: 24 | OMT: n: 27 | CPAP: n: 27 | Combined: n: 22
Brazil				
				Sex: 100% male | Age: 25-65 years (mean 48.1 SD: 11.2)
Van Dyck et al., 2016^(18)^	Children undergoing treatment with myofunctional therapy	To investigate the effects of OMT on the tongue behavior of children with anterior open bite	22	No expansion (OMT): n: 6 | Age: 7.1-10.6 years (average 8.3 SD: 0.8)
Belgium				
				No expansion (Non-OMT): n: 4 | Age: 7.1-10.6 years (average 9.1 SD: 1.2)
				With expansion (OMT): n: 6 | Age: 7.1-10.6 years (average 8.4 SD: 0.3)
				With expansion (Non-OMT): No: 6 | Age: 7.1-10.6 years (average 8.7 SD: 0.9)
				Sex: -
Prado et al., 2018^(19)^	Adults, orthognathic surgery	To determine the effect of OMT in individuals with dentofacial deformity undergoing orthognathic surgery	48	Treatment: n: 13 | Age: 18-45 years (mean 29.31 SD: 8.87)
Brazil				
				Non-treatment: n: 10 | Age: 18-45 years (mean 28.10 SD: 5.30)
				Sex: -
Huang et al., 2019^(20)^	Children and adolescents with OSAHS	To conduct a prospective study in randomized, age-matched children undergoing myofunctional therapy or a functional device used during sleep	110	OMT: n: 54 | Age: 4-16 years (average 7.02 SD: 2.44)
United States				
				Sex: 27 (50%) man, 27 (50%) women
				Night device: n: 56 | Age: 4-16 years (average 7.97 SD: 3.08)
				Sex: 36 (64%) male, 20 (36%) female
Van den Steen et al., 2018^(21)^	Healthy elderly	To investigate the effect of anterior and posterior tongue strengthening exercises on tongue strength and measure possible detraining effects	16	Group anterior exercise: n: 9 | Age: 75-95 years (average 84 years)
Belgium				
				Group posterior exercise: n: 7 | Age: 75-95 years (average 84 years)
				Sex: 8 male and 8 female
Byeon, 2018^(22)^	Patients who were diagnosed with flaccid dysarthria due to stroke	To identify the effects of the tongue-pressure exercise protocol and the traditional orofacial exercise on the articulation muscle and percentage of correct consonants of the patients with dysarthria	21	Control group: n: 11 | Age: - (average 67.03 SD 7.6)
South Korea				
				Sex: 10 male and 5 female
				Treatment group: n: 10 | Age: - (average 65.85 SD 9.23)
				Sex: 11 male and 6 female
Baudelet et al., 2020^(23)^	Adults undergoing chemotherapy and the presence of dysphagia	To investigate the effect of specific adherence measures on patients’ actual compliance, wellbeing, muscle strength, swallowing function and quality of life during and following (chemo)radiotherapy	-	Registration of the study protocol
Belgium				
O'Connor-Reina et al., 2020^(24)^	Patients with severe obstructive sleep apnea-hypopnea syndrome	To evaluate the effects of the new mobile health (mHealth) app in patients with severe obstructive sleep apnea-hypopnea syndrome	28	Control group: n: 10 | Age: - (median 63.9)
				Sex: 2 (20%) female
				AirwayGym group: n: 18 | Age: - (median 59.17)
Spain				Sex: 4 (22%) female
Poncin et al., 2022^(25)^	Adults diagnosed with moderate obstructive sleep apnoea	To assess the effects of a 6 weeks tongue elevation training programme in patients with obstructive sleep apnoea		Control group: n: 13 | Age: - (Median 56.0)
Belgium				
				Sex: 6 (46%) males
				Therapy group: n: 12 | Age: - (Median 48.0)
				Sex: 8 (66%) males
Baudelet et al., 2023^(26)^	Adults undergoing chemotherapy and the presence of dysphagia	To investigate the effect of 3 different service-delivery modes on actual patients’ adherence.	148	Paper group: n: 49 | Age: Average: 63 SD: 9.5
Belgium				
				Sex: 14 (29%) female and 35 (71%) male
				App group: n: 49 | Age: Average: 63 SD: 7.9
				Sex: 11 (22%) female and 38 (78%) male
				Therapist supported: n: 50 | Age: 63 Average: 63 SD: 8.2
				Sex: 10 (20%) female and 40 (80%) male

**Caption:** OSAHS: Obstructive Sleep Apnea Hypopnea Syndrome; OMT: Orofacial Myofunctional Therapy; CPAP: Continuous Positive Airway Pressure; n: number of participants; SD: standard deviation; NSOMExs: Non-speech oro-motor exercises.

Regarding the nomenclature and description of the executed exercises, eight studies described these two items in a sufficiently clear manner, generating no doubt from the reviewers about the used exercises^(11,13,16,21-23,25,26)^. Six studies did not inform how long the contraction was maintained during exercise^(9,17-20,24)^. Four studies presented the exercises’ name, but did not describe their execution^(9,11,19,24)^; and two did not present the exercises’ name and description^(18,20)^ (Table 3).

**Table 3:** Exercises of tongue, execution, and prescribed parameters.

Author/Year	Exercise of tongue: execution	Series	Repetitions	Duration Isometry Rest	Time in therapy	Frequency
Degan and Puppin-Rontani, 2005^(9)^	- Counter-resistance with wooden spatula: without further information	-	-	30 min therapy	8 weeks	1 time a week
	- Tongue snapping on the palate: without further information					
Guimarães et al., 2009^(10)^	- Palate sweep (anterior-posterior): tip of the tongue on the anterior palate and slide to the posterior	-	-	3 min daily each exercise	3 months	Daily
	- Tongue sucked on the palate: press the tongue on the palate					
	- Force the back of the tongue on the oral floor: while keeping the tip on the lower incisors					
Carnaby-Mann et al., 2012^(11)^	- Placebo: “Valchuf” buccal extension maneuver	4	10	10 min	6 months	2 times a day
	- High-intensity: tongue pressure, swallowing with effort					
Kothari et al., 2011^(12)^	- Tongue-protrusion task in the laboratory	-	288	1h and two 1h sessions	-	-
	- Standard session: 288 repeated and identical trials					
	- Modulation session: 1h of tongue-training with modulation of training parameters every 20 min (3 × 96 trials with different settings – A, B, C)					
Clark, 2012^(13)^	- Strength: anterior elevation of the tongue to 100% of the recent measurement	5	Strength: 5	3 to 10 min - rest from 60 to 90 s	4 weeks	3 times a week
			Resistance: 75% of the previous			
	- Resistance: increase before 50% of the recent measure. Number of repetitions calculated at 75% of the most recent					
			Power: 10			
	- Power: the IOPI light came on at the target pressure (75%). Sound / t / as soon as possible					
	- Speed: Sound / t / as fast as possible.					
Lazarus et al., 2014^(14)^	- Retraction, elevation, protrusion, and lateralization of the tongue	10	-	2s	6 weeks	5 times a day
	- Isometric exercises: press the tongue against a depressor for 2 s in the direction previously instructed					5 times a week
Mackenzie et al., 2014^(15)^	Repetitions of tongue and lip movements which had relevance to positions for speech sounds and tongue elevation behind the upper teeth	-	5	5s	8 weeks	2 to 3 times
				with rest		5 times a week
Nuffelen et al., 2015^(16)^	Tongue pressure: press the tongue against the palate for 3s	12	5	3s	2 weeks 24 sessions	3 times a week
Diaféria et al., 2017^(17)^	- push the tip of the tongue against the hard palate and slide the tongue backward (A)	-	A: 20 times B: 20 times C: 10 times right and left side D: 20 times	20 min (x3= 60 min per day)	3 months	3 times a day
	- suck the tongue upward against the palate, pressing the entire tongue against the palate (B)					
	- tongue rotation in the oral vestibule (C)					
	- forcing the back of the tongue against the floor of the mouth while keeping the tip of the tongue in contact with the inferior incisive teeth (D)					
Van Dyck et al., 2016^(18)^	Without further information	-	-	-	4-6 months	1 to 2 times a week
					10-20 sessions	
Prado et al., 2018^(19)^	- Mobility exercises (isotonic)	-	-	-	10 weeks	1 time a week
	- Exercises for tone					
Huang et al., 2019^(20)^	- Without further information	-	-	20 min	1 year	Daily
Van den Steen et al., 2018^(21)^	- Tongue pressure against the bulb.	24	120	3s sustaining contraction	8 weeks	3 times a week on non-consecutive days
				5 - 30s of rest after each repetition		
				Levels recalculated every 2 weeks according to the progressive overload.		
Byeon, 2018^(22)^	- Raising the tip of the tongue: With closing the lips, raise the tip of the tongue as much as possible and maintain it for 5 seconds. Conduct it with opening the lips.	4	5	5s sustaining contraction	4 weeks	1 time a day 5 times a week
	- Raising the tip of the tongue with overcoming the resistance: With opening the lips, raise the tip of the tongue as much as possible with resisting the pressure of a tongue depressor and maintain it for 5 seconds.					
	- Moving the tongue left and right: With closing the lips, move the tongue left and right and maintain each pose for 5 seconds. Conduct it with opening the lips.					
	- Moving the tongue left and right with overcoming the resistance: With closing the lips, move the tongue left and right with resisting the pressure of a tongue depressor. Maintain each pose for 5 seconds.					
	- Pushing the tongue out: With closing the lips, push the tongue as straight as possible and as far as possible and hold the pose for five seconds. Conduct it with opening the lips.					
	- Pushing the tongue out with overcoming the resistance: With opening the lips, push the tongue as straight as possible and as far as possible resisting the pressure of a tongue depressor. Hold the pose for five seconds.					
Baudelet et al., 2020^(23)^	- Chin tuck: counter-resistance target level from 60 to 70% of 1RM	Strength: 12 Chin tuck: 30	Strengthening: 10	Strengthening: 30s of rest between sets	4 weeks	5 times a week
	- Deglutition with effort					
	- Tongue strengthening: target level set at 80% of 1RM					
			Chin tuck: 5 - 1 swallowing with effort after 5	Swallowing with effort: 3s		
	- The IOPI instrument was used in the swallowing exercise					
O'Connor-Reina et al., 2020^(24)^	- 9 exercises based on myofunctional therapy that are aimed is to increase the tone of the extrinsic muscles of the tongue (genioglossus, hyoglossus, styloglossus, and palatoglossus)^A^	-	-	20 min	3 months	Daily
Poncin et al., 2022^(25)^	-Strength task: tongue against the hard palate to squeeze the IOPI bulb positioned immediately posterior to the central incisors	A: 3 B: 3 C: 3 D: 4 E: 4 F: 4 G: 2 H: 3	A: 10 B: 10 C: 10 D: 12 E: 12 F: 12	Isometric pressure: 2s Rest: 2min	6 weeks	1 time a day and 4 times a week
	Week 1: Load 60% (A)					
	Week 2: Load 65% (B)					
	Week 3: Load 70% (C)					
	Week 4: Load 70% (D)					
	Week 5: Load 75% (E)					
	Week 6: Load 80% (F)					
	- Endurance task: to maintain an isometric lingual pressure on the IOPI bulb equivalent to 50% of the baseline strength value until task failure, for more than 2s					
	Week 1 to 3: 50% (G)					
	Week 4 to 6: 50% (H)					
Baudelet et al., 2023^(26)^	- Chin tuck: counter-resistance target level from 60 to 70% of 1RM	Strength: 12 Chin tuck: 30	Strengthening: 10 Chin tuck: 5 - 1 swallowing with effort after 5	Strengthening: 30s of rest between sets Swallowing with effort: 3s	4 weeks	5 times a week
	- Deglutition with effort					
	-Tongue strengthening: target level set at 80% of 1RM					
	- The IOPI instrument was used in the swallowing exercise					

^A^: The study says that the exercises were based on those described by Guimarães et al.^(10)^

**Caption:** min: minute; s: second, IOPI: Iowa Oral Performance Instrument; RM: repetition maximum

Regarding the exercises’ parameters, six studies presented the number of sets, repetitions and muscle contraction time^(11,16,22,23,25,26)^; two studies did not present the muscle contraction time^(10,17)^ and one did not present the number of repetitions^(14)^. Eight studies did not present these parameters sufficiently for the exercises to be replicated^(9,10,12,14,18-20,24)^ (Table 3).

As for the exercises’ goals and the results evaluation, three studies did not clearly define the exercises’ objectives^(11,12,24)^. Objective assessments of the tongue musculature were used in twelve studies^(11-14,16,18,21-26)^. The Iowa Oral Performance Instrument (IOPI), a portable instrument connected to a small balloon filled with air, which transmits to the digital display the isometric pressure (in kilopascals, kPa) that the tongue produces when pressing the balloon against the palate, was used in ten studies^(13,14,16,18,21-26)^. Objective assessments, but not directly of the tongue musculature, were used in four studies^(10,17,19,20)^. One study described only the protocols used^(9)^. The combination of clinical assessment with IOPI was mentioned in two studies^(16,18)^ (Table 4).

**Table 4:** Description of the exercise objective, method used for the evaluation of the tongue and main results

Author/Year	Purpose of the exercise	Evaluation of tongue	Main results
Degan and Puppin-Rontani, 2005^(9)^	Isometric and counter-resistance	Felício Protocol (1999): Lingual positioning and swallowing of water and food	OMT showed better tongue positioning and more adequate swallowing pattern
Guimarães et al., 2009^(10)^	Isotonic and isometric	Does not describe specific assessment for tongue	TG: significant decrease in neck circumference, frequency and intensity of snoring, daytime sleep, sleep quality score and OSAHS severity
Carnaby-Mann et al., 2012^(11)^	-	Magnetic resonance imaging (genioglossus, mylohyoid and hyoglossus)	Mylohyoid, genioglossus and hyoglossus had greater deterioration in the control group
Kothari et al., 2011^(12)^	-	Tongue force transducer	All participants improved performance during 60 min of standard tongue-training and the ability to roll the tongue did not influence tongue-training performance
			In the standard session there was a main effect of time and there was no main effect of sequence
Clark., 2012^(13)^	Isotonic and isometric	IOPI	Initial evidence that the specificity of the training can be observed in the tongue musculature
Lazarus et al., 2014^(14)^	Isotonic and isometric	IOPI	With no significant difference in tongue strength and oropharyngeal swallowing efficiency between groups, quality of life related to speaking, eating and social life improved in both
Mackenzie et al., 2014^(15)^	Isometric	Four protocols were used to evaluate speech, lips, and tongue	The inclusion of non-speech oro-motor exercises did not appear to influence outcomes
Nuffelen et al., 2015^(16)^	Isometric	IOPI and protocols	In progress
Diaféria et al., 2017^(17)^	Isotonic and isometric	Myofunctional assessment	The OTM + CPAP group showed greater adherence to CPAP. Therapy can be considered an adjunctive treatment and a strategy for adhering to CPAP
Van Dyck et al., 2016^(18)^	Isotonic and isometric	IOPI, tongue position, swallowing	Myofunctional therapy influenced the behavior of the tongue
Prado et al., 2018^(19)^	Isotonic, isometric, and functional training	Felício et al. Protocol (2010): position at rest and volume	Positive effects of therapy on clinical and electromyographic aspects
Huang et al., 2019^(20)^	Isotonic and isometric	Does not describe specific assessment for tongue	Great abandonment of therapy. Compared to 6 months, some aspects were better in the device group, sleep latency was better in the therapy group
Van den Steen et al., 2018^(21)^	Isometric	IOPI	Training for the anterior part resulted in greater strength than for the posterior
Byeon, 2018^(22)^	Isometric	IOPI	The combined rehabilitation program improved the maximal tongue strength and maximal lip strength. However, there was no difference in the correct articulation between the two groups
Baudelet et al., 2020^(23)^	Isometric	IOPI and dynamometer	Registration of the study protocol
O'Connor-Reina et al., 2020^(24)^	-	IOPI	The severity of symptoms decreased, and the tone of the upper airway muscles increased after 3 months
Poncin et al., 2022^(25)^	Isotonic and isometric	IOPI	In the control group, only tongue force significantly improved. In the therapy group, tongue force and endurance as well as subjective sleepiness, quality of sleep and fatigue significantly improved
Baudelet et al., 2023^(26)^	Isometric	IOPI and dynamometer	The aim of the study was not to verify the results in the participants' tongue

**Caption:** OMT: orofacial myofunctional therapy; OSAHS: Obstructive Sleep Apnea Hypopnea Syndrome; CPAP: continuous positive airway pressure; IOPI: Iowa Oral Performance Instrument; TG: therapy group

### Methodological Quality

The PEDro Scale analysis shows that, methodologically, studies have a good score. The Scale warns that the high score does not directly provide evidence of the clinical usefulness of the treatment. In addition, it should not be used to compare studies from different therapy areas, especially since in some areas of physiotherapy it is not possible to satisfy all items on the scale^(8)^. The methodology regarding randomization, blinding and analysis of study results was quite satisfactory. However, despite the good methodological quality of the studies, other scales need to be created that assess the technical quality of the different exercises and training programs in obtaining the desired clinical results.

In the PEDro Scale analysis, two studies were excluded^(16,23)^, as they are related to the clinical trial registration and not research results. Since the objective of this review was not to verify the exercises’ results, but rather what exercises were used, we decided to keep these studies in the review, but they were not evaluated through PEDro for not compromising the analysis score in the items related to the results. The average score of the sixteen studies analyzed was 6.9, with an SD of 1.36. The most frequent scores were 7 (31.25%) and 6 (31.25%), and 56.3% of the studies scored ≥7. All studies specified the eligibility criteria (although this item is not used for the score), and presented the measures’ precision and variability, statistical data, and groups similarity. The items random allocation in groups^(12,21)^, secret allocation^(12,21,22)^ and intention to treat^(19)^ were not described in these studies. One study specified blinded participants^(25)^ and two indicated therapist blinding^(9,25)^ (Table 5).

**Table 5:** PEDro scale analysis

Study	PEDro Scale Criteria											
	Specified Eligibility Criteria*	Random Allocation	Blind Allocation	Similar Groups	Blinded Participants	Blinded Therapist	Blinded Reviewer	Results >85%	Intent to Treat	Statistical Comparisons	Accuracy and Variability Measures	Total
Degan and Puppin-Rontani, 2005^(9)^	Y	Y	Y	Y	N	Y	Y	Y	Y	Y	Y	9
Guimarães et al. 2009^(10)^	Y	Y	Y	Y	N	N	N	Y	Y	Y	Y	7
Carnaby-Mann et al., 2012^(11)^	Y	Y	Y	Y	N	N	Y	Y	Y	Y	Y	8
Kothari et al., 2011^(12)^	Y	N	N	Y	N	N	N	Y	Y	Y	Y	5
Clark, 2012^(13)^	Y	Y	Y	Y	N	N	N	Y	Y	Y	Y	7
Lazarus et al., 2014^(14)^	Y	Y	Y	Y	N	N	N	N	Y	Y	Y	6
Mackenzie et al., 2014^(15)^	Y	Y	Y	Y	N	N	Y	Y	Y	Y	Y	8
Diaféria et al., 2017^(17)^	Y	Y	Y	Y	N	N	Y	N	Y	Y	Y	7
Van Dyck et al., 2016^(18)^	Y	Y	Y	Y	N	N	N	N	Y	Y	Y	6
Prado et al., 2018^(19)^	Y	Y	Y	Y	N	N	N	Y	N	Y	Y	6
Huang, et al., 2019^(20)^	Y	Y	Y	Y	N	N	N	N	Y	Y	Y	6
Van den Steen et al., 2018^(21)^	Y	N	N	Y	N	N	N	Y	Y	Y	Y	5
Byeon, 2018^(22)^	Y	Y	N	Y	N	N	N	Y	Y	Y	Y	6
O'Connor-Reina et al., 2020^(24)^	Y	Y	Y	Y	N	N	N	Y	Y	Y	Y	7
Poncin et al., 2022^(25)^	Y	Y	Y	Y	Y	Y	Y	Y	Y	Y	Y	10
Baudelet et al., 2023^(26)^	Y	Y	Y	Y	N	N	N	Y	Y	Y	Y	7

* "Specified Eligibility Criteria" does not score

**Caption:** Y = Yes, N = No;

## DISCUSSION

On one hand, the fact that 18 randomized clinical trials^(9-26)^ were found in the existing literature suggests that the idea of a lack of publications dealing with exercises for the tongue musculature in the speech therapy does not hold true. Of the 18 studies, 13 were published in the last ten years, and only three were developed on a healthy population. On the other hand, the fact that only 18 studies were included, and that it was not possible to carry out a meta-analysis due to their methodological diversity, shows that studies with higher methodological quality are still needed to better elucidate the tongue anatomy and its function, as well as which exercises are the most appropriate to train/rehabilitate this musculature^(29)^.

While reviewing the existent literature, we observed a lack of uniformity in the nomenclature of the prescribed exercises, a lack of standardization of the prescriptions and a lack of uniformity in the evaluated parameters. Studies in healthy populations are needed to determine normality standards or normal values for the outcomes, which can then be used to guide evidence-based clinical practice on what is a healthy condition, and to better define which outcomes are suitable for the orofacial muscles according to the different goals. Once the parameters and their objectives are defined, they should be applied to different populations (e.g. the different facial growth patterns) as there is also a lack of studies with excellent methodological quality and that have used clear training parameters for the musculature of the tongue in these different populations.

A study, designed to verify the students' knowledge about the commonly prescribed exercises, while enrolled in their final year of full-time study in either a Bachelor of Speech Pathology or Masters of Speech Pathology Program, demonstrated that they did not master the appropriate use of exercises in different areas of speech therapy. The authors suggest that discussing and deepening the knowledge in this area would be beneficial for students and clinical supervisors^(30)^, in order to define a clear methodology, with a clear description of the exercises, as well as clearly identifying the parameters that should be used in different training programs for this musculature. Without a uniform nomenclature, and an adequate description of exercises and training parameters, it is difficult to clearly define which exercises are effective, what their effects are, and which training program would be the most suitable for the treatment of different orofacial disorders in clinical practice. The need for greater knowledge about exercise physiology was also verified in a study with trained professionals; the authors also indicate the need for more technical-scientific support to guide clinical practice^(31)^.

Eight studies described the exercises clearly enough and presented a number of sets, repetitions and muscle contraction time^(11,13,16,21-23,25,26)^, whereas ten studies did not disclose such parameters, making it impossible to replicate the exercises^(9,10,12,14,15,17-20,24)^. The strength training strategies for large muscle groups in the body has its parameters quite consolidated. On one hand, strength training requires the use of high loads to increase muscle force production capacities and improve the muscle structure and fiber quality^(32)^. On the other hand, resistance training depends on the number of repetitions and sets imposed during training as the main variables^(33)^. Muscle power, which decreases in large muscles with aging, benefits from training programs that vary the speed of contraction and the used loads^(34)^.

However, orofacial muscles are recruited differently and have different neuromuscular structure than large skeletal muscles responsible to generate motion. Therefore, the orofacial myofunctional therapy, being a science that also intervenes in the skeletal musculature, needs to consolidate its parameters according to the desired objectives, aimed at producing the necessary adaptations to the intrinsic characteristics of its treated musculature. In addition, muscle loading is an incipient theme in speech therapy. Strength training at high intensities leads to the adaptation (i.e., hypertrophy) of type II muscle fibers. In the context of training, strength usually refers to the maximal strength and is measured during a single or a minimum number of repetitions^(13)^. Although there are different ways and different equipment available to apply loads to limb muscles, orofacial muscles bring a challenge due to the difficulty in implementing similar methods as those used for limb muscles^(13,32-34)^.

Nevertheless, IOPI is the instrument worldwide used for assessing tongue pressure/strength^(35)^. Such an instrument, or another with similar function, is needed for the strength training principles (e.g., loads and overload) to be applied. Without a clear definition of which loads, or which mechanical overload should be applied for strengthening orofacial muscles in rehabilitation, it becomes difficult to evaluate the effects of different exercise programs, as well as to clearly define which parameters should be used for the treatment of each neuromuscular dysfunction.

Despite the importance of the tongue for our most basic daily living activities (e.g., eating, speaking), the understanding of its structure and function apparently is still at an early stage. An explanation for the lack of studies on the human tongue could be due to its complex anatomy. There are few anatomical resources in the literature clearly showing this complex anatomy, and this has constituted a real barrier for researchers in this field. Thus, the diagnosis and treatment of tongue disorders are delayed in relation to other structures of the head and neck^(2)^. Since the tongue is a muscular hydrostat, it differs from other skeletal muscle groups, as its movement is performed by a complex pattern of contractions of fibers aligned in intersecting planes. In addition, this structure does not move around a joint. Thus, the morphological and biomechanical properties of the tongue and its supporting musculature differ substantially from the skeletal musculature of the limbs and core. Therefore, one question regarding the existing knowledge in the strength-training field is how the training specificity would manifest itself in this muscle group. At the present time, knowledge is still limited to indicate whether the tongue muscle group shows specificity effects similar to those of the limbs, also because initial investigations were not striking^(13)^.

The limitations of this research are the design of the studies considered. This research included only clinical trials due to methodological rigor and because they are considered clinical studies with the highest level of evidence, but relevant studies with other designs may not have been identified. This research did not perform a manual search, outside of the systematic search. It is observed that some studies use descriptors not present in MESH, which prevents them from being located in the databases with Boolean operators with MESH terms. Our study verified the need, which has also been pointed out by other authors^(30,31)^, for research that evaluates the effectiveness of prescribed exercises considering different parameters in a healthy population and in populations with different pathologies.

## CONCLUSIONS

For the question it was proposed to answer, this review found that the parameters used in the exercises prescribed by speech therapists in the oromyofunctional rehabilitation of the tongue musculature varied widely. There is no shortage in the literature of exercise prescriptions, and there is a good quality of the reviewed studies. However, there is a lack of consensus and of a clear description of the exercises’ goals, as well as a lack of a clear description of the parameters indicated for achieving specific rehabilitation goals. This can lead to confusion and inadequate exercise prescription in clinical practice. Therefore, there is a need for studies, with objective measures, aimed at defining, according to specificity on strength, endurance, power, and speed, which are the effects of different parameters on this musculature both in healthy subjects and in orofacial patients of different populations. Such concepts need to be better understood and applied to the reality of orofacial myofunctional therapy of the tongue musculature.
